# Understanding The Mimicker: Epidemiological Pattern and Determinant of Melioidosis Mortality in Negeri Sembilan, Malaysia

**DOI:** 10.1371/journal.pntd.0012147

**Published:** 2024-05-06

**Authors:** Shahrul Azhar Md Hanif, Mohd Rohaizat Hassan, Muhammad Ridzwan Rafi’i, Ahmad Farid Nazmi Abdul Halim, Mohd ‘Ammar Ihsan Ahmad Zamzuri, Muhammad Ismail, Siti Salwa Ibrahim, Massitah Mihat, Lokman Rejali, Muhammad Habiruddin Zubir, Muhammad Salihin Mahadi, Shazwanis Ahmad Ismail, Veshny Ganesan, Muhammad Fahmi Mohd Fadzil

**Affiliations:** 1 Department of Public Health Medicine, Faculty of Medicine, National University of Malaysia Jalan Yaacob Latif, Bandar Tun Razak, Cheras, Kuala Lumpur, Malaysia; 2 Borneo Medical and Health Research Centre, Faculty of Medicine and Health Sciences, Universiti Malaysia Sabah, Kota Kinabalu, Sabah, Malaysia; 3 State Health Department of Negeri Sembilan, Ministry of Health Malaysia, Jalan Rasah, Seremban, Negeri Sembilan, Malaysia; 4 Department of Community Health, Universiti Putra Malaysia, Serdang, Malaysia; 5 Universiti Malaysia Sabah, Jalan UMS, Kota Kinabalu, Sabah, Malaysia; 6 Seremban District Health Office, Jalan Lee Sam, Seremban, Negeri Sembilan, Malaysia; University of Florida, UNITED STATES

## Abstract

**Background:**

Melioidosis, a tropical infectious disease caused by *Burkholderia pseudomallei*, is epidemic in most region in Southeast Asia with high case fatality. However, there is scanty information regarding the disease’s epidemiological pattern, demographics, and underlying risk factors.

**Method:**

This 5-year retrospective study of 185 confirmed cases which were taken from the Negeri Sembilan Melioidosis Registry between 2018 and 2022. We aim to describe the incidence, mortality rate, case fatality, relationship with meteorology, and factors that influence mortality in this central region of Peninsular Malaysia.

**Results:**

Incidence rate (IR) of melioidosis in Negeri Sembilan is varied at 1.9 to 5.1 with mean of 3.1 in 100,000 population per year. IR varied between districts in the state from zero to 22.01 in 100,000 population per year. Mortality rate were ranged from 0.17 to 0.74 cases with mean of 0.44 cases in 100,000 population per year. The case fatality rate of this state scattered from 8.70% to 16.67%. There were no significant linear associations between cases and deaths with monthly rainfall and humidity. The mean age of patients was 52.8 years, predominated with age around 41–60 years old. Males (77.8%) predominated, and the majority of cases were Malays (88.9%) and had exposed to soil related activities (74.6%). Mortality from melioidosis was more likely in Bumiputera and non-Malaysians (*p*<0.05). Patients who had at least one comorbidity were at a higher risk of death from melioidosis (*p*<0.05). Diabetes mellitus was found in 41.1% of all identified cases, making it a major underlying risk factor for both developing and dying from melioidosis (aOR:19.32, 95%CI:1.91–195.59, *p*<0.05). Hypertension and mortality status in melioidosis are also significantly correlated (aOR: 7.75, 95% CI: 2.26–26.61, *p*<0.05).

**Conclusion:**

The epidemiological patterns of cases reported from Negeri Sembilan are consistent for the most part from previous studies in other states in Malaysia and global with regard to its incidence, case fatality, demographic and predisposing chronic diseases. Diabetes mellitus and hypertension were significantly linked to increased mortality among all determinants.

## Author summary

Melioidosis is an environmentally acquired infection caused by *Burkholderia pseudomallei*, with the global distribution of this bacteria is comparatively restricted to temperate zones. The details on epidemiological pattern, demographics, and underlying risk factors of the disease, however, are still scarce. This study examines a 5-year retrospective analysis of 185 confirmed cases that were obtained between 2018 and 2022 from the Negeri Sembilan Melioidosis Registry. In this central region of Peninsular Malaysia, the authors detailed the incidence, death rate, case fatality, correlation with meteorology, and factors influencing mortality. Findings indicated that the state had a 3.1 in 100,000 population yearly mean incidence rate of melioidosis, with a case fatality rate ranging from 8.70% to 16.67%. The incidences vary throughout districts. Nevertheless, no significant relationships were found between monthly humidity and rainfall and the number of cases and fatalities. The main factors found to be associated with a higher mortality rate included bumiputra ethnicities, underlying comorbidities and diabetes mellitus. Remarkably, there was also a correlation found between the mortality status in melioidosis with hypertension. Despite a few limitations, these findings could contribute to improving healthcare professionals’ knowledge of the illness and for better prevention care.

## Introduction

Melioidosis is a tropical infectious disease caused by a gram-negative aerobic bacillus called *Burkholderia pseudomallei* (*B*. *pseudomallei*) which usually presents in soil and water. This bacterium’s global distribution is relatively limited to temperate zones based on documented incidences of melioidosis. However, it is considered endemic in most region in Southeast Asia, South Asia, Australia, and South America. In developing tropical countries, melioidosis has been regarded as a potential emerging infectious disease that can be possible threat in the region [1]. According to a study model, there were about 165,000 cases of melioidosis in 2015 among the 3 billion people who lived in the locations most likely to harbour *B*. *pseudomallei*, with an annual incidence rate of 5 per 100,000 persons at risk [1]. East Asia and the Pacific region are anticipated to have 40% of the world’s melioidosis cases. The case fatality rate (CFR) of melioidosis is 10–50% [2]. The predicted mortality from melioidosis is around 89,000 individuals per year, on par with other high priority infections such as measles (95,600 individuals per year) and higher than that for leptospirosis (50,000 individuals per year) and dengue infection (12,500 individuals per year) [1].

Melioidosis has been presence in Malaysia since early 1920s. It does not fall under mandatory notifiable disease in Prevention and Control of Infectious Disease Act 1988 (Act 342). For that reason, the true prevalence and incidence of melioidosis in Malaysia remain unclear, although cases keep spiking with more than hundred death cases recorded throughout the states [3]. The hotspot of this disease however located on states with high agricultural activities. Scattered reports from Malaysia showed the incidence rate of melioidosis in Sabah, Pahang and Kedah states were 3.6, 6.1 and 16.35 per 100,000 populations per year respectively with no true mortality rate [4]. CFR is inconsistent between states such as 12.3% in Sarawak, Kelantan (33%), and Kedah (34%). [5–7].

In Malaysia, multiple studies concluded the impact of rainfall, pre-existing medical disorders, occupational exposure, underlying diabetes mellitus, older age and male are the among melioidosis risk factors that lead to mortality in melioidosis [3,4,7]. However, the melioidosis pattern are varied among the states in Malaysia due to the climate and sociodemographic difference. Further understanding of the epidemiological characteristic of melioidosis in the population are necessary for appropriate preventive and control measures to reduce annual incidence and mortality. Thus, the aim of this study is to determine the epidemiological pattern and risk factors associated with mortality in melioidosis in Negeri Sembilan, Malaysia.

## Material and method

### Ethics statement

Since the study only using secondary data, the requirement for written consent was waived by the Medical Review and Ethics Committee Ministry of Health Malaysia. Permission for publication was granted by Ministry of Health (NIH.800-4/4/1 Jld 124 (24)) in view of the data owned by national surveillance system.

This study was carried out in Negeri Sembilan, a state located in the central part of Peninsular Malaysia. It was a retrospective review based on the data report of melioidosis patients from Negeri Sembilan State Melioidosis Registry from year 2018 to 2022. Melioidosis is not a notifiable disease nationally. However, it is notifiable disease in this state, thus all clinically suspected and confirmed case are captured by the surveillance system through reporting from mainly government and private hospital. All cases included in this study as most independent variables were completely written. Diagnosis of cases were made based on the Ministry of Health Infectious Disease Case Definition (2017). Confirmed cases are based on either by culture or serology. Bacterial cultures were obtained by inoculating clinical specimens onto rich media, blood agar and/or chocolate agar and/or MacConkey agar. For blood cultures, BACTEC 9240 Instrumented Blood Culture System was used for confirmation. *B*. *pseudomallei* was identified using the API20NE (Analytical Profile Index, Non-Enterobacteriaceae) fast identification system. Serology was based on the highly specific and sensitive Indirect Fluorescent Antibody (IFA) test.

The component of reporting and documentation are based on variables and categories available in the registry format. Demographic details and variables related to factors associated with melioidosis infection were collected from the database. This includes age, gender, race/ethnicity, occupation, exposure risk factors and underlying comorbidities. Comorbidities of interest were later defined and separated into diabetes mellitus, hypertension, and renal failure, based on the few studies literatures [4,8]. The total of confirmed and death melioidosis cases from January 2018 to December 2022 were used in this study.

Incidence rates were calculated by dividing total melioidosis cases reported over total population for each respected year, whilst total cases of melioidosis deaths were divided by the population for each relevant year to determine mortality rates. Total estimated population for each year were obtained from Department of Statistics Malaysia (DOSM). The incidence and mortality rates were reported per 100,000 population. Case fatality for every year were determined by percentages of melioidosis death cases over total melioidosis cases of the respective year. Average monthly rainfall, monthly rainfall days and average monthly humidity meteorological data for Negeri Sembilan were plotted using data available from 2018 to 2022 by Malaysian Meteorological Department. Kuala Pilah, one of the districts in this state were chosen as reference for meteorological data as it captures most of the weather readings throughout the year of study and a focal point of data in this state. Association between these meteorological data with number of melioidosis cases were analysed using Pearson correlation.

The normality of the data was investigated graphically (using a histogram and a Q-Q plot) and statistically (using skewness, kurtosis, and Shapiro-Wilks/Kolmogorov-Smirnov statistics). Percentage is used to summarize categorical socio-demographic and underlying comorbidities variables. Chi square and Fisher’s exact test were used to compare associations between each independent variables with disease outcomes. The independent variables included were tested. Logistic regression analysis was used to further analyse the confounding factors. SPSS version 23 (SPSS Inc., Chicago, IL, USA) was used for all statistical analyses and *p* < 0.05 was considered as statistically significant.

## Result

Table 1 shows the incidence rate (IR), mortality rates and case fatality of melioidosis between year 2018 to 2022. The annual incidence rates of melioidosis in Negeri Sembilan varied from 1.91 to 5.08 in 100,000 population with mean of 3.1 cases in 100,000 annually. IR between districts in this state scattered around no cases to 22.0 cases in 100,000 population. Jempol, located at eastern of this state recorded the highest mean incidence rate annually with 9.9 in 100,000 population and registered highest incidence rate of 22 cases in 100,000 population in 2020. Southwest part of this state, Port Dickson recorded the lowest mean incidence rate per year with around 1.4 cases in 100,000 population. As overall mortality rate of Negeri Sembilan, it narrowly ranged from 0.17 to 0.74 cases with mean of 0.44 cases in 100,000 population per year. The case fatality rate of this state scattered from 8.70% to 16.67%.

**Table 1:** Annual Incidence Rate, Mortality Rate and Case Fatality Rate of Melioidosis in Negeri Sembilan.

	Year				
	2018	2019	2020	2021	2022
**Incidence Rate (IR)**					
Negeri Sembilan	1.98	2.03	5.08	1.91	4.47
**IR by Districts**					
Jelebu	2.2	0	8.68	0	6.49
Jempol	0.79	5.51	22.01	3.93	17.27
Kuala Pilah	2.88	7.16	5.69	7.14	8.58
Port Dickson	2.34	1.56	2.33	0	0.77
Rembau	2.12	0	2.11	0	4.19
Seremban	1.75	1.16	2.31	1.44	2.14
Tampin	2.29	2.28	5.68	3.38	5.62
**Mortality Rate**					
Negeri Sembilan	0.26	0.34	0.67	0.17	0.74
**Case Fatality Rate (%)**					
Negeri Sembilan	12.5	16.67	13.33	8.70	16.67

Incidence and mortality rate per 100,000 population

**Fig 1:**
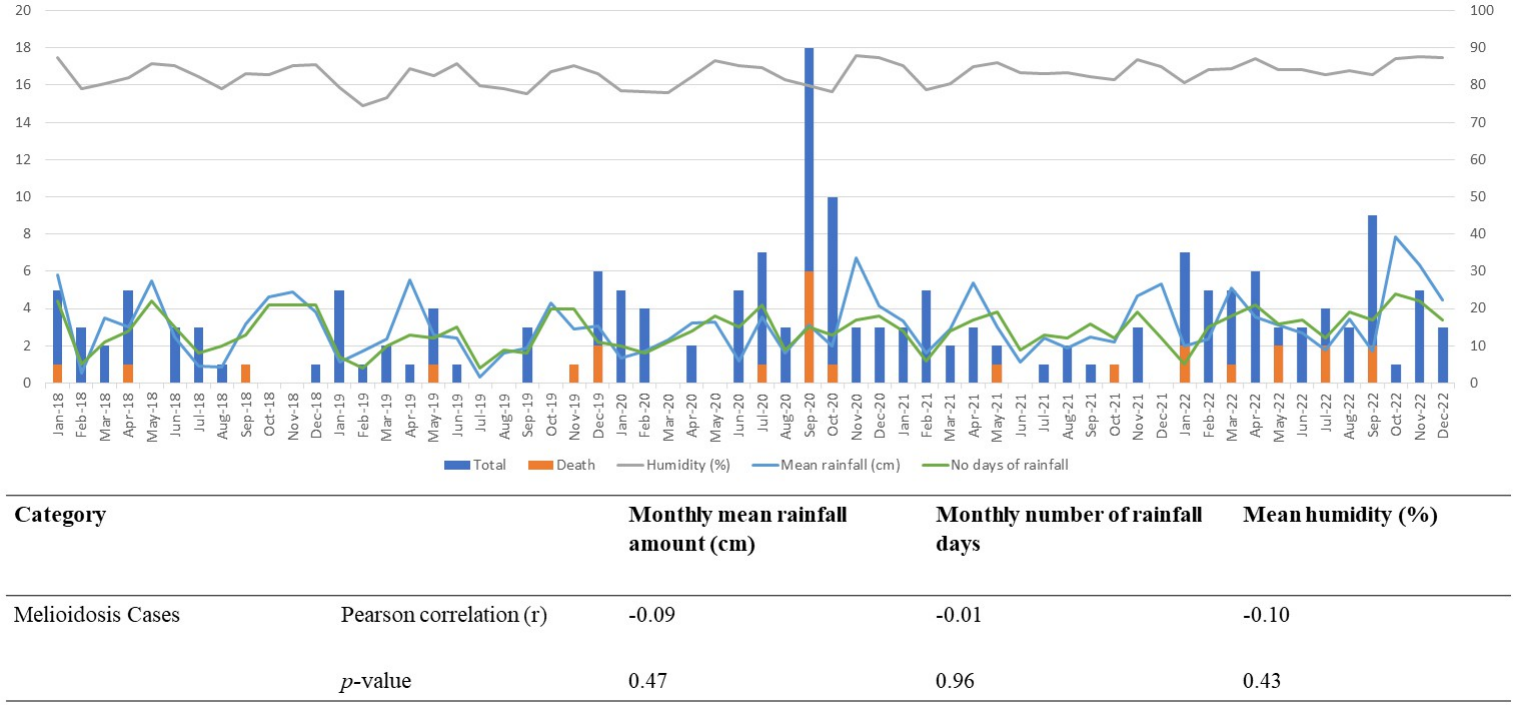
Association of number of melioidosis cases in relation to humidity, mean rainfall and days of rainfall.

Fig 1 depicts association between the occurrence of melioidosis cases and in relation to meteorological factors which are the observed mean monthly rainfall amount, number of rainfall days per month and humidity in Negeri Sembilan. From early 2018 through the end of 2019, there were consistently less than six cases of melioidosis and fewer than two deaths monthly. In 2020, there were increment of melioidosis cases and death, which spiking around September and October of 2020, then gradually declined to less than six cases each month. There was occasional spike of cases (below 10 cases) in 2022. The average monthly caseload from 2018 through 2022 were around 3.1 cases. The mean rainfall amount throughout these five years is around 15.3 cm per month, with average 14 rainfall days. Humidity in this state ranging around 74% to 88% with mean of 82% per month. The plots demonstrate that disease cases were not affected by the wettest month. Most of highest cases recorded happen in dry spell with rainfall amount less than 20cm and 15 rainfall days, with humidity maintain more than 80% throughout the year. Pearson correlation shows no association between average monthly mean rainfall amount (r = -0.09, *p* = 0.47), monthly rainfall days (r = -0.01, *p* = 0.96) and average monthly humidity (r = -0.10, *p* = 0.43) in relation to melioidosis cases.

A total of 185 cases of melioidosis were recorded during throughout 2018 to 2022 (Table 2). A total of 182 cases were confirmed with blood culture, and only 3 cases with serology. The distribution of observed cases was distinctly linear throughout the population of study. The mean age of patients was 52.8 years (range: 9–103 years), with almost half of cases ranging at 41–60 years old (51.4%). Only three patients (1.6%) were aged < 18 years. Males (144 patients; 77.8%) predominated melioidosis cases and death recorded in this state. Most of the melioidosis cases were unemployed or retiree (78%). In term of exposure risk factor, notably 138 cases (74.6%) of the recorded cases had exposed to soil related activities such as gardening, farming, and soil-related works. However, there were no statistical significance between age, gender, occupation, and exposure risk factor in relation to survival status in this population The ethnicity of patients however showed significant association with survival status (p = 0.019). Malay ethnicity recorded most cases (131 patients;70.8%), however in proportion to death, Bumiputera and other ethnicities which include migrant workers shows more notable findings with six death (46.2%) out of 13 cases (OR:6.63, 95%CI:1.97–22.36, *p*<0.05).

**Table 2:** Socio-demographic Characteristics and Underlying Diseases of Patients and Associations with Survival Status.

Variable	Alive (*n* = 159) *n* (%)	Death (*n* = 26) *n* (%)	Total (N)-185) (%)	*p-*value	OR (95% CI)
**Socio-demographic Characteristics**					
**Age group**					
1–40 years old	30 (85.7)	5 (14.3)	35 (18.9)	0.189^b^	1.0
41–60 years old	78 (82.1)	17 (17.9)	95 (51.4)		1.31 (0.44,3.86)
>60 years old	51 (92.7)	4 (7.3)	55 (29.7)		0.47 (0.12,1.89)
**Gender**					
Female	37 (90.2)	4 (9.8)	41 (22.2)	0.369^b^	1.0
Male	122 (84.7)	22 (15.3)	144 (77.8)		1.67 (0.54,5.15)
**Race/Ethnicity**					
Malay	116 (88.5)	15 (11.5)	131 (70.8)	<0.05^b^	1.0
Bumiputra/Others*	7 (53.8)	6 (46.2)	13 (7.0)		6.63 (1.97,22.36)
Indian	27 (87.1)	4 (12.9)	31 (16.8)		1.15 (0.35,3.73)
Chinese	9 (90.0)	1 (10.0)	10 (5.4)		0.86 (0.10,7.23)
**Occupation**					
Transport services	8 (88.9)	1 (11.1)	9 (4.8)	0.951^b^	1.0
Unemployed/Retiree	68 (87.2)	10 (12.8)	78 (42.2)		1.18 (0.13,10.43)
Agricultural/Farming	35 (83.3)	7 (16.7)	42 (22.7)		1.60 (0.17,14.9)
Factory/Industry	19 (90.5)	2 (9.5)	21 (11.4)		0.84 (0.07,10.7)
Professionals	16 (84.2)	3 (15.8)	19 (10.3)		1.5 (0.13,16.8)
Self-employed	13 (81.3)	3 (18.8)	16 (8.6)		1.85 (0.16,20.93)
**Exposure Risk Factor**					
Non-soil related	43 (91.5)	4 (8.5)	47 (25.4)	0.236^b^	1.0
Soil-related	116 (84.1)	22 (15.9)	138 (74.6)		2.04 (0.66,6.26)
**Underlying Diseases**					
**Comorbidities**					
No Comorbidity	78 (91.8)	7 (8.2)	85 (45.9)	<0.05^a^	1.0
At least 1 Comorbidity	81 (81.0)	19 (19.0)	100 (54.1)		2.61 (1.04,6.56)
**Diabetes Mellitus Status**					
Non-Diabetic	102 (93.6)	7 (6.4)	109 (58.9)	<0.05^a^	1.0
Diabetic	57 (75.0)	19 (25.0)	76 (41.1)		4.86 (1.93,12.25)
**Hypertension Status**					
Non-Hypertension	133 (91.1)	13 (8.9)	146 (78.9)	<0.05^a^	1.0
Hypertension	26 (66.7)	13 (33.3)	39 (21.1)		5.12 (2.13,12.29)
**Renal Failure Status**					
No Renal Failure	155 (86.1)	25 (13.9)	180 (97.3)	0.535^b^	1.0
Chronic Renal Failure	4 (80.0)	1 (20.0)	5 (2.7)		1.55 (0.17,14.44)

^a^Chi-square Test

^b^Fisher’s Exact Test

*Bumiputra/Others: Includes indigenous people (Orang Asli), Sabah natives and non-Malaysian

For underlying comorbidities, half of the observed cases (54.1%) had at least one comorbidity which includes diabetes mellitus (DM), hypertension, renal failure and other chronic diseases. Around 19 (19.1%) cases of those with comorbidities lost their lives to melioidosis and it is statistically significant (OR:2.61, 95%CI:1.04–6.56, *p*<0.05). DM only constituted 76 cases (41.1%) of total recorded cases throughout these five years. However, patients with DM suffered significantly more mortality from melioidosis compared to those who did not have this risk factor (OR:4.86, 95%CI:1.93–12.25, *p*<0.05). From this registry, only 39 cases (21.1%) had hypertension. In relation to survival status, around 33.3% of patient with hypertension accounted for melioidosis death compared to those with no hypertension (8.9%) and were observed to be a significant predictor (OR:5.12, 95%CI:2.13–12.29, *p*<0.05). Chronic renal failure recorded only 5 cases (2.7%) and was not significantly associated with the survival status of the patients. Other diseases occurred in a very few numbers of patients in this population to be assume statistical importance.

Table 3 shows the multiple logistic regression analysis results of potential associated factors survival status in melioidosis. Only diabetes mellitus and hypertension were identified as significant survival status in melioidosis. Patients with DM had 19.32 higher odds of melioidosis mortality compared to those without DM (aOR:19.32, 95%CI:1.91–195.59, *p*<0.05). The odds of melioidosis mortality in hypertensive patients were 7.75 times higher than in non-hypertensive (aOR:7.75, 95% CI:2.26–26.61, *p*<0.05).

**Table 3:** Factors Associated with Mortality Status in Melioidosis (using multiple logistic regression model).

Variable	OR (95% CI)	Wald	aOR (95% CI)	ꭕ2 stat (df)^a^	*p*-value^b^
**Age group**					
1–40 years old	1.0		1.0		
41–60 years old	1.31 (0.44,3.86)	0.24	1.67 (0.49,5.73)	0.67 (1)	0.412
>60 years old	0.47 (0.12,1.89)	1.13	0.29 (0.06,1.43)	2.31 (1)	0.129
**Comorbidities**					
No Comorbidity	1.0		1.0		
At least 1 Comorbidity	2.61 (1.04,6.56)	4.20	0.09 (0.01,1.02)	3.77 (1)	0.052
**Diabetes Mellitus Status**					
Non-Diabetic	1.0		1.0		
Diabetic	4.86 (1.93,12.25)	11.21	19.32 (1.91,195.59)	6.29 (1)	<0.05
**Hypertension Status**					
Non-Hypertension	1.0		1.0		
Hypertension	5.12 (2.13,12.29)	13.33	7.75 (2.26,26.61)	10.60 (1)	<0.05

aOR = adjusted odds ratio,

^a^ Wald test,

^b^*p*<0.05 indicates a significant statistical result

No interaction between variables, No multicollinearity, No influential outlier

Hosmer Lemeshow goodness of fit test not significant (p = 0.784)

Sensitivity, specificity of model prediction = 87.6%

R^2^: 0.3

## Discussion

The mean incidence of melioidosis recorded for Negeri Sembilan is comparable with other states in Malaysia which ranging from 2.57 to 16.35 [9]. The incidence is almost equivalent with incidence rate of neighbouring country such as Singapore (0.6–2.4 per 100,000) and Thailand (3.95 per 100,000) [10,11]. Globally, it is predicted that there would be 165,000 cases worldwide in 2015, with an incidence rate of 5.0 per 100,000 persons being at risk each year [1]. The incidence in region where melioidosis is prevalent can reach up to 50 per 100,000 annually [8]. The difference in incidence rates by district may be due to the difference in geographical conditions, sociodemographic and socioeconomic makeup of the population in Negeri Sembilan. The incidences were observed to be much higher in agricultural driven areas compared to industrial and manufacturing driven areas. More urbanized area such as Seremban and Port Dickson recorded mean incidence less than 2 in 100,000 persons annually compared to Jempol and Kuala Pilah with incidence around 6 to 9.9 in 100,000 persons. It is consistent with the findings observed in Sarawak where marked variation between districts ranging from 5.8 to 29.3 per 100,000 [12]. Rural districts had higher incidences of melioidosis compared to urban and main districts. The exact reason behind the surge in incidence of melioidosis in the year 2020 is unknown. It can be suggested potential causes include a shift in the pattern of referrals from clinics and hospitals, awareness and an increase in the prevalence of comorbidities [13]. Another possible reason is the change from the pandemic COVID-19 Movement Control Order (MCO) to the Conditional Movement Control Order (CMCO) and Recovery Movement Control Order (RMCO), which expedites the operation of the agricultural and industrial sectors in order to recoup economic losses. A change in attention towards the peak of COVID-19 cases in Malaysia in 2021 may have contributed to underdiagnosis or underreporting, which might also explain the abrupt decline in incidence during that year.

Case fatality in this state is averaging at 14% annually. It is considered low when compared to other states in Malaysia which ranging from 33% to 54% regardless of its bacteraemia status [4]. However, the case fatality is comparable with global data which falls within the range of 10%-50%. The mean mortality rate of this state is approximately 4 in 1 million persons annually, which is below global estimation [8]. In northeast Thailand, Australia, and the Northern Territory, the annual incidence of melioidosis is up to 50 cases per 100,000 people [1]. Multiple factors affecting the case fatality and mortality rate in this study. These might be accounted for by the rising awareness of this disease, early recognition, driven on by better diagnostic predictions and early treatment in the study population [4]. The observed diversity in mortality between other region may be primarily caused by differences in the proportion of presenting and related diseases among sick populations. The prevalence of diabetes mellitus, hypertension, chronic alcohol consumption and other related significant predisposing factors of melioidosis are different between population/region. Other factors that need to be taken into account in the variation of mortality rates include host genetic susceptibility for acquiring melioidosis, geographic strain differences in the pathogenicity and virulence of the bacteria, and variations in both pathogen and host responses to conventional treatment regimens [2].

Melioidosis is an environmentally acquired infection. The occurrence of its causative agent can be associated with presence of animals, changes in composition and physicochemical changes in soil, and the climate variability. Thus, weather change influence both the distribution of and annual incidence of melioidosis. Established previous studies concluded a significant association of rainfall and humidity with melioidosis, especially on rate and mode of transmission of infection [2]. However, we found no significant correlation of melioidosis cases in Negeri Sembilan with total rainfall, and humidity levels. The findings consistent with reports in Kuala Lumpur and Singapore [14,15]. The study population is unaffected by the weather change since the distribution of patients is more concentrated in urban areas, even though soil and water are the primary means of acquisition of this disease. In this state’s industrialised and urbanised areas, exposure to soil and water is uncommon [16]. Due to the persistently high humidity throughout the year (mean 82% monthly) and fluctuation in rainfall volume (1.6cm to 40cm) throughout the distribution of cases in Negeri Sembilan, it is difficult to compare and link melioidosis and weather change in this study. A false fallacy could result from generalising the association throughout the entire state based on a single district’s focus point of meteorological data.

The melioidosis patients’ characteristics in Negeri Sembilan match those described in other areas. Nevertheless, a strong correlation between melioidosis mortality and Bumiputra (other than Malays) and other ethnic groups was found (46.2% case fatality, p<0.05). In Malaysia, the name "Bumiputra" refers to the Malays, the Orang Asli of Peninsular Malaysia, and the different indigenous groups of East Malaysia. In this study’s population of Bumiputra and other ethnicities, there are also indigenous people (Orang Asli), and 5 migrant workers. This group has a higher propensity for melioidosis mortality because of its geographic (rural) location and high exposure to soil and water due to the active agricultural activity. [17,18]. Socioeconomic disadvantages especially among indigenous people such as household income, education level and healthcare access do play a role in determining the outcome of melioidosis. As such, a study conducted in Australia found that socioeconomic disadvantages of these group raise the likelihood of developing melioidosis [19]. In another study by Arushothy et al., it demonstrates that clinical isolates of *B*. *pseudomallei* are genetically diverse among states and ethnic groups in Malaysia, which may be another factor influencing the severity of melioidosis [20]. Additionally, a possible relationship between sequence types and antibiotic resistance was also reported.

An interesting conclusion from this study is that people with diabetes mellitus have a higher probability of developing melioidosis than those without the disease, making diabetes the most prevalent predisposing factor for the disease. In this investigation, we discovered that people with diabetes have 19 more odds of dying from melioidosis than people without diabetes. This is greater than the estimated relative risk determined by Chowdury et al.’s systematic review and meta-analysis, which ranged from 1.5 to 13.1 [21]. Melioidosis among individuals with diabetes mellitus may occur due to poor management of glycemic levels and impaired innate immunity in diabetic patients. Comparatively to melioidosis cases without diabetes mellitus, acute melioidosis cases with diabetes mellitus exhibited a suppressed cellular adaptive immune response. Poor glycaemic management has been linked to infectious disorders in several studies, and the link between infection and higher haemoglobin A1c (HbA1c) levels is well known [22,23]. As diabetes mellitus is prevalent in Malaysia, a thorough strategy and approach to improve diabetes control, prevention, and treatment is important to reduce melioidosis mortality in the state [24].

Another noteworthy finding from our study is that we found a significant association of hypertension with mortality status in melioidosis. In this study, one-fifth of the study population had hypertension (21.1%) which is almost equivalent is most of other studies in Malaysia. However, multivariate logistic regression analysis revealed that hypertension individuals had a 7.8-fold higher risk of dying from melioidosis than non-hypertensive patients. The result caught our attention because there haven’t been much research linking the mortality from melioidosis to this non-communicable disease. Any infectious disease mortality may be influenced by elevated blood levels of C-reactive protein, which raise the risk of coagulopathy and increase capillary permeability especially in hypertensive patients [25]. There is a possibility that poorly controlled hypertension, which then causes cardiovascular events, is responsible for the mortality in people with hypertension. The impact of hypertension to cardiac hemodynamic and structure, impaired fibrinolysis, endothelial dysfunction, and thrombosis become the factor of Intensive Care Unit admission and disease mortality [26].

It is noteworthy that the data from other states/region and our studies are based on better notification, ascertainment, and reporting of disease cases. Thus, it implies that the calculated average annual incidence is probably representative of the actual incidence of melioidosis in our research population and comparable with other studies. However, there is still a possibility of underreporting as melioidosis is not a mandatory notification under Prevention and Control of Infectious Diseases Act 1988. The mimic characteristics of melioidosis with other disease such as tuberculosis increase the chances of underreporting [27]. The registry data lacks information on clinical presentation, laboratory and radiological test results, characterization of bacteria isolates and treatment to clinical outcomes. A comprehensive set of data will provide proper association and validation of clinical predictors of mortality, thus helping in developing a proper epidemiological surveillance, priority investigations, antibiotics susceptibility testing and treatment initiation. Since serological tests are neither sensitive nor specific for melioidosis, the registry should only accept confirmed cultures as verification of the disease [2]. Future research studies evaluating the epidemiological and environmental risk factors behind the disease’s natural history utilising standardised data from many perspectives are necessary. As such, analysing the link between different time lags after disease onset and the humidity and rainfall volume using a regression model may lead to a more favourable association.

## Conclusion

The mean incidence of melioidosis in Negeri Sembilan is almost at par with that in other states of Malaysia. However, contrasted to other states, the mortality rate and case fatality observed were significantly lower. Meteorological factors such as humidity and rainfall were found to be non-significant with melioidosis cases reported. The main factors found to be associated with a higher mortality rate included bumiputra ethnicities, underlying comorbidities, underlying diabetes mellitus and hypertension. These discoveries might be helpful in enhancing healthcare professionals’ understanding of the illness and their capacity to create awareness, diagnose, prompt treatment and intervention effectively. Only by acknowledging the existence of melioidosis as a significant tropical disease will new understanding and the development of effective therapies emerge.

## Supporting information

S1 Fig Melioidosis cases in relation to humidity, mean rainfall and days of rainfall. (DOCX)

S1 Table Sociodemographic characteristics and underlying disease of melioidosis cases. (DOCX)

## References

[1] Limmathurotsakul D, Golding N, Dance DA, Messina JP, Pigott DM, Moyes CL, et al. Predicted global distribution of Burkholderia pseudomallei and burden of melioidosis. Nat Microbiol. 2016;1(1). doi: 10.1038/nmicrobiol.2015.8 27571754

[2] Cheng AC, Currie BJ. Melioidosis: epidemiology, pathophysiology, and management. Clin Microbiol Rev. 2005;18(2):383–416. doi: 10.1128/CMR.18.2.383-416.2005 15831829 PMC1082802

[3] Hadi FS, Ghazali S, Ahmad N, Ramli SR. Trend and pattern of melioidosis seropositivity among suspected patients in Malaysia 2015–2019. Trop Biomed. 2021;38(4):561–7. doi: 10.47665/tb.38.4.099 35001922

[4] Nathan S, Chieng S, Kingsley PV, Mohan A, Podin Y, Ooi MH, et al. Melioidosis in Malaysia: Incidence, Clinical Challenges, and Advances in Understanding Pathogenesis. Trop Med Infect Dis. 2018;3(1). doi: 10.3390/tropicalmed3010025 30274422 PMC6136604

[5] Toh V, Tee SP, Lee S-H. Clinical characteristics and predictors of mortality in patients with melioidosis: the Kapit experience. Tropical Medicine & International Health. 2021;26(6):664–71. doi: 10.1111/tmi.13563 33590932

[6] Zueter A, Yean CY, Abumarzouq M, Rahman ZA, Deris ZZ, Harun A. The epidemiology and clinical spectrum of melioidosis in a teaching hospital in a North-Eastern state of Malaysia: a fifteen-year review. BMC Infect Dis. 2016;16:333. doi: 10.1186/s12879-016-1583-2 27423906 PMC4947242

[7] Hassan MRA, Pani SP, Peng NP, Voralu K, Vijayalakshmi N, Mehanderkar R, et al. Incidence, risk factors and clinical epidemiology of melioidosis: a complex socio-ecological emerging infectious disease in the Alor Setar region of Kedah, Malaysia. BMC Infectious Diseases. 2010;10(1):302. 20964837 10.1186/1471-2334-10-302 PMC2975659

[8] Wiersinga WJ, Virk HS, Torres AG, Currie BJ, Peacock SJ, Dance DAB, et al. Melioidosis. Nat Rev Dis Primers. 2018;4:17107. doi: 10.1038/nrdp.2017.107 29388572 PMC6456913

[9] Tang RY, Lim SH, Lam JE, Nurasykin S, Eileen T, Chan YW. A 5-year retrospective study of melioidosis cases treated in a district specialist hospital. Med J Malaysia. 2019;74(6):472–6. 31929471

[10] Sim SH, Ong CEL, Gan YH, Wang D, Koh VWH, Tan YK, et al. Melioidosis in Singapore: Clinical, Veterinary, and Environmental Perspectives. Trop Med Infect Dis. 2018;3(1). doi: 10.3390/tropicalmed3010031 30274428 PMC6136607

[11] Hantrakun V, Kongyu S, Klaytong P, Rongsumlee S, Day NPJ, Peacock SJ, et al. Clinical Epidemiology of 7126 Melioidosis Patients in Thailand and the Implications for a National Notifiable Diseases Surveillance System. Open Forum Infect Dis. 2019;6(12):ofz498. doi: 10.1093/ofid/ofz498 32083145 PMC7020769

[12] Sia TLL, Mohan A, Ooi MH, Chien SL, Tan LS, Goh C, et al. Epidemiological and Clinical Characteristics of Melioidosis Caused by Gentamicin-Susceptible Burkholderia pseudomallei in Sarawak, Malaysia. Open Forum Infect Dis. 2021;8(10):ofab460. doi: 10.1093/ofid/ofab460 34646909 PMC8500297

[13] Limmathurotsakul D, Wongratanacheewin S, Teerawattanasook N, Wongsuvan G, Chaisuksant S, Chetchotisakd P, et al. Increasing incidence of human melioidosis in Northeast Thailand. Am J Trop Med Hyg. 2010;82(6):1113–7. doi: 10.4269/ajtmh.2010.10-0038 20519609 PMC2877420

[14] Heng BH, Goh KT, Yap EH, Loh H, Yeo M. Epidemiological surveillance of melioidosis in Singapore. Ann Acad Med Singap. 1998;27(4):478–84. 9791650

[15] Sam IC, Puthucheary SD. Melioidosis and rainfall in Kuala Lumpur, Malaysia. J Infect. 2007;54(5):519–20. doi: 10.1016/j.jinf.2006.07.007 16965821

[16] Aburas M, Ho Y, Ramli M, Ashaari Z. Evaluating Urban Growth Phenomena in Seremban, Malaysia, Using Land-Use Change-Detection Technique. International Postgraduate Conference on Agriculture, Environmental and Waste Management (IPCAEWM 2015). 2015.

[17] Chakravorty A, Heath C. Melioidosis: An updated review. Australian Journal for General Practitioners. 2019;48:327–32. doi: 10.31128/AJGP-04-18-4558 31129946

[18] Hii SFF, Kee CC, Ahmad N. Melioidosis: Overview of seropositivity in Malaysia. Trop Biomed. 2016;33(4):697–701. 33579066

[19] Hanson J, Smith S, Stewart J, Horne P, Ramsamy N. Melioidosis—a disease of socioeconomic disadvantage. PLOS Neglected Tropical Diseases. 2021;15(6):e0009544. doi: 10.1371/journal.pntd.0009544 34153059 PMC8248627

[20] Arushothy R, Amran F, Samsuddin N, Ahmad N, Nathan S. Multi locus sequence typing of clinical Burkholderia pseudomallei isolates from Malaysia. PLOS Neglected Tropical Diseases. 2021;14(12):e0008979. 10.1371/journal.pntd.0008979 PMC7793247 33370273

[21] Chowdhury S, Barai L, Afroze SR, Ghosh PK, Afroz F, Rahman H, et al. The Epidemiology of Melioidosis and Its Association with Diabetes Mellitus: A Systematic Review and Meta-Analysis. Pathogens. 2022;11(2). doi: 10.3390/pathogens11020149 35215093 PMC8878808

[22] Daryabor G, Atashzar MR, Kabelitz D, Meri S, Kalantar K. The Effects of Type 2 Diabetes Mellitus on Organ Metabolism and the Immune System. Front Immunol. 2020;11:1582. doi: 10.3389/fimmu.2020.01582 32793223 PMC7387426

[23] Hirji I, Guo Z, Andersson SW, Hammar N, Gomez-Caminero A. Incidence of urinary tract infection among patients with type 2 diabetes in the UK General Practice Research Database (GPRD). J Diabetes Complications. 2012;26(6):513–6. doi: 10.1016/j.jdiacomp.2012.06.008 22889712

[24] Akhtar S, Nasir JA, Ali A, Asghar M, Majeed R, Sarwar A. Prevalence of type-2 diabetes and prediabetes in Malaysia: A systematic review and meta-analysis. PLOS ONE. 2022;17(1):e0263139. doi: 10.1371/journal.pone.0263139 35085366 PMC8794132

[25] Mehta P, Hotez PJ. NTD and NCD Co-morbidities: The Example of Dengue Fever. PLoS Negl Trop Dis. 2016;10(8):e0004619. doi: 10.1371/journal.pntd.0004619 27561091 PMC4999202

[26] Su S, Chen R, Zhang S, Shu H, Luo J. Immune system changes in those with hypertension when infected with SARS-CoV-2. Cell Immunol. 2022;378:104562. doi: 10.1016/j.cellimm.2022.104562 35901625 PMC9183242

[27] Singh M, Mahmood M. Melioidosis: the great mimicker. J Community Hosp Intern Med Perspect. 2017;7(4):245–7. doi: 10.1080/20009666.2017.1348875 29046753 PMC5637701

