# Supplementary material for: Understanding The Mimicker: Epidemiological Pattern and Determinant of Melioidosis Mortality in Negeri Sembilan, Malaysia
Source: PLoS Negl Trop Dis. 2024 May 6;18(5):e0012147. doi: 10.1371/journal.pntd.0012147 (PMC11098469; doi:10.1371/journal.pntd.0012147)
Supplement: S1 Fig — (DOCX) [file pntd.0012147.s001.docx]

S1 Melioidosis cases in relation to humidity, mean rainfall and days of rainfall

| Month | Total | Death | Humidity (%) | Mean rainfall (cm) | No days of rainfall |
| --- | --- | --- | --- | --- | --- |
| Jan-18 | 5 | 1 | 87.40323 | 29.06 | 22 |
| Feb-18 | 3 | 0 | 78.93571 | 2.6 | 5 |
| Mar-18 | 2 | 0 | 80.33 | 17.44 | 11 |
| Apr-18 | 5 | 1 | 81.97667 | 15.04 | 14 |
| May-18 | 0 | 0 | 85.64194 | 27.54 | 22 |
| Jun-18 | 3 | 0 | 85.17333 | 12.38 | 15 |
| Jul-18 | 3 | 0 | 82.2871 | 4.68 | 8 |
| Aug-18 | 1 | 0 | 79.02903 | 4.2 | 10 |
| Sep-18 | 1 | 1 | 83.09259 | 15.78 | 13 |
| Oct-18 | 0 | 0 | 82.71379 | 23.08 | 21 |
| Nov-18 | 0 | 0 | 85.28333 | 24.44 | 21 |
| Dec-18 | 1 | 0 | 85.53871 | 19.08 | 21 |
| Jan-19 | 5 | 0 | 79.20645 | 5.62 | 7 |
| Feb-19 | 1 | 0 | 74.37778 | 8.6 | 4 |
| Mar-19 | 2 | 0 | 76.67 | 11.9 | 10 |
| Apr-19 | 1 | 0 | 84.31667 | 27.73 | 13 |
| May-19 | 4 | 1 | 82.44839 | 12.94 | 12 |
| Jun-19 | 1 | 0 | 85.68333 | 12.02 | 15 |
| Jul-19 | 0 | 0 | 79.88065 | 1.58 | 4 |
| Aug-19 | 0 | 0 | 79.16774 | 8.08 | 9 |
| Sep-19 | 3 | 0 | 77.58333 | 9.34 | 8 |
| Oct-19 | 0 | 0 | 83.63226 | 21.44 | 20 |
| Nov-19 | 1 | 1 | 85.21667 | 14.51 | 20 |
| Dec-19 | 6 | 2 | 83.2129 | 15.41 | 11 |
| Jan-20 | 5 | 0 | 78.50323 | 6.62 | 10 |
| Feb-20 | 4 | 0 | 78.28276 | 8.72 | 8 |
| Mar-20 | 0 | 0 | 78.08333 | 11.54 | 11 |
| Apr-20 | 2 | 0 | 82.19565 | 16.08 | 14 |
| May-20 | 0 | 0 | 86.54194 | 16.48 | 18 |
| Jun-20 | 5 | 0 | 85.12 | 5.96 | 15 |
| Jul-20 | 7 | 1 | 84.75806 | 17.8 | 21 |
| Aug-20 | 3 | 0 | 81.52258 | 8.12 | 9 |
| Sep-20 | 18 | 6 | 79.93667 | 15.72 | 15 |
| Oct-20 | 10 | 1 | 78.21935 | 9.96 | 13 |
| Nov-20 | 3 | 0 | 88.03 | 33.64 | 17 |
| Dec-20 | 3 | 0 | 87.27419 | 20.66 | 18 |
| Jan-21 | 3 | 0 | 85.28065 | 16.58 | 14 |
| Feb-21 | 5 | 0 | 78.73929 | 8.04 | 6 |
| Mar-21 | 2 | 0 | 80.45161 | 14.66 | 14 |
| Apr-21 | 3 | 0 | 84.99 | 26.78 | 17 |
| May-21 | 2 | 1 | 86.03871 | 15.02 | 19 |
| Jun-21 | 0 | 0 | 83.28333 | 5.76 | 9 |
| Jul-21 | 1 | 0 | 83.10645 | 12.2 | 13 |
| Aug-21 | 2 | 0 | 83.24194 | 9.52 | 12 |
| Sep-21 | 1 | 0 | 82.32667 | 12.32 | 16 |
| Oct-21 | 1 | 1 | 81.5871 | 11 | 12 |
| Nov-21 | 3 | 0 | 86.82333 | 23.48 | 19 |
| Dec-21 | 0 | 0 | 84.86129 | 26.56 | 12 |
| Jan-22 | 7 | 2 | 80.76774 | 10.08 | 5 |
| Feb-22 | 5 | 0 | 84.26071 | 11.76 | 15 |
| Mar-22 | 5 | 1 | 84.44839 | 25.6 | 18 |
| Apr-22 | 6 | 0 | 87.16333 | 17.68 | 21 |
| May-22 | 3 | 2 | 84.04516 | 15.7 | 16 |
| Jun-22 | 3 | 0 | 84.26 | 13.48 | 17 |
| Jul-22 | 4 | 2 | 82.72258 | 8.98 | 12 |
| Aug-22 | 3 | 0 | 83.93226 | 17.22 | 19 |
| Sep-22 | 9 | 2 | 82.84667 | 8.62 | 17 |
| Oct-22 | 1 | 0 | 87.21613 | 39.16 | 24 |
| Nov-22 | 5 | 0 | 87.60667 | 31.62 | 22 |
| Dec-22 | 3 | 0 | 87.46129 | 22.38 | 17 |
