# Supplementary material for: Understanding The Mimicker: Epidemiological Pattern and Determinant of Melioidosis Mortality in Negeri Sembilan, Malaysia
Source: PLoS Negl Trop Dis. 2024 May 6;18(5):e0012147. doi: 10.1371/journal.pntd.0012147 (PMC11098469; doi:10.1371/journal.pntd.0012147)
Supplement: S1 Table — (DOCX) [file pntd.0012147.s002.docx]

S1 Table Sociodemographic characteristics and underlying disease of melioidosis cases

| AGE RANGE | RACE | OCCUPATION | STATUS | BLOOD TEST | EXPOSURE RISK FACTOR | COMORBIDITIES | Diabetes Mellitus (DM) Status | Hypertension (HPT) Status | Renal Failure (RF) Status |
| --- | --- | --- | --- | --- | --- | --- | --- | --- | --- |
| 1-40 | Indian | Professionals | Alive | Culture and Sensitivity | Non-soil exposed | 1 comorbid and more | DM | no HPT | no RF |
| >60 | Malay | Unemployed | Alive | Culture and Sensitivity | Non-soil exposed | 1 comorbid and more | DM | HPT | RF |
| 1-40 | Indian | Professionals | Alive | Culture and Sensitivity | Non-soil exposed | No comorbid | no DM | no HPT | no RF |
| 41-60 | Malay | Factory/Industry | Alive | Culture and Sensitivity | Soil exposed | 1 comorbid and more | no DM | no HPT | no RF |
| >60 | Malay | Agricultural/Farming | Alive | Culture and Sensitivity | Soil exposed | 1 comorbid and more | DM | HPT | no RF |
| 41-60 | Malay | Unemployed | Alive | Culture and Sensitivity | Non-soil exposed | No comorbid | no DM | no HPT | no RF |
| 1-40 | BumiputeraOthers | Factory/Industry | Alive | Culture and Sensitivity | Non-soil exposed | 1 comorbid and more | DM | no HPT | no RF |
| 41-60 | Malay | Agricultural/Farming | Alive | Culture and Sensitivity | Soil exposed | No comorbid | no DM | no HPT | no RF |
| 41-60 | Malay | Unemployed | Alive | Culture and Sensitivity | Non-soil exposed | No comorbid | no DM | no HPT | no RF |
| 1-40 | Malay | Factory/Industry | Alive | Culture and Sensitivity | Soil exposed | No comorbid | no DM | no HPT | no RF |
| >60 | Indian | Professionals | Alive | Culture and Sensitivity | Soil exposed | No comorbid | no DM | no HPT | no RF |
| >60 | Indian | Factory/Industry | Alive | Culture and Sensitivity | Non-soil exposed | 1 comorbid and more | DM | HPT | no RF |
| 41-60 | Indian | Unemployed | Death | Culture and Sensitivity | Soil exposed | 1 comorbid and more | DM | HPT | no RF |
| >60 | Malay | Unemployed | Alive | Culture and Sensitivity | Non-soil exposed | 1 comorbid and more | no DM | HPT | no RF |
| >60 | Malay | Agricultural/Farming | Alive | Culture and Sensitivity | Soil exposed | 1 comorbid and more | no DM | no HPT | no RF |
| >60 | Malay | Unemployed | Alive | Serology | Soil exposed | 1 comorbid and more | no DM | no HPT | no RF |
| >60 | Malay | Unemployed | Alive | Culture and Sensitivity | Non-soil exposed | No comorbid | no DM | no HPT | no RF |
| 41-60 | Malay | Unemployed | Alive | Culture and Sensitivity | Non-soil exposed | 1 comorbid and more | DM | no HPT | no RF |
| 41-60 | Chinese | Factory/Industry | Death | Culture and Sensitivity | Soil exposed | 1 comorbid and more | DM | no HPT | RF |
| 41-60 | Malay | Factory/Industry | Alive | Culture and Sensitivity | Soil exposed | 1 comorbid and more | DM | no HPT | no RF |
| 41-60 | Indian | Unemployed | Alive | Culture and Sensitivity | Non-soil exposed | 1 comorbid and more | no DM | no HPT | no RF |
| 41-60 | Indian | Factory/Industry | Alive | Culture and Sensitivity | Soil exposed | No comorbid | no DM | no HPT | no RF |
| 1-40 | BumiputeraOthers | Agricultural/Farming | Death | Culture and Sensitivity | Soil exposed | 1 comorbid and more | DM | HPT | no RF |
| 41-60 | Malay | Factory/Industry | Alive | Culture and Sensitivity | Soil exposed | 1 comorbid and more | no DM | HPT | no RF |
| 41-60 | BumiputeraOthers | Agricultural/Farming | Alive | Culture and Sensitivity | Soil exposed | No comorbid | no DM | no HPT | no RF |
| 41-60 | Malay | Services | Alive | Culture and Sensitivity | Non-soil exposed | 1 comorbid and more | DM | no HPT | no RF |
| 41-60 | Malay | Professionals | Alive | Culture and Sensitivity | Non-soil exposed | 1 comorbid and more | DM | no HPT | no RF |
| 41-60 | Indian | Services | Alive | Culture and Sensitivity | Soil exposed | No comorbid | no DM | no HPT | no RF |
| >60 | Malay | Unemployed | Alive | Culture and Sensitivity | Soil exposed | No comorbid | no DM | no HPT | no RF |
| 1-40 | Malay | Unemployed | Alive | Serology | Non-soil exposed | No comorbid | no DM | no HPT | no RF |
| 41-60 | Malay | Services | Alive | Culture and Sensitivity | Soil exposed | No comorbid | no DM | no HPT | no RF |
| >60 | Malay | Unemployed | Alive | Culture and Sensitivity | Soil exposed | No comorbid | no DM | no HPT | no RF |
| 41-60 | BumiputeraOthers | Unemployed | Alive | Culture and Sensitivity | Soil exposed | 1 comorbid and more | DM | HPT | no RF |
| >60 | Malay | Agricultural/Farming | Alive | Culture and Sensitivity | Soil exposed | 1 comorbid and more | DM | HPT | no RF |
| >60 | Malay | Agricultural/Farming | Alive | Culture and Sensitivity | Soil exposed | 1 comorbid and more | DM | no HPT | no RF |
| 41-60 | Malay | Unemployed | Death | Culture and Sensitivity | Soil exposed | 1 comorbid and more | DM | HPT | no RF |
| 41-60 | Indian | Self-employed | Alive | Culture and Sensitivity | Non-soil exposed | 1 comorbid and more | DM | no HPT | no RF |
| 41-60 | Indian | Professionals | Alive | Culture and Sensitivity | Soil exposed | 1 comorbid and more | DM | no HPT | no RF |
| 41-60 | Malay | Factory/Industry | Alive | Culture and Sensitivity | Soil exposed | 1 comorbid and more | DM | HPT | no RF |
| 41-60 | Chinese | Factory/Industry | Alive | Culture and Sensitivity | Non-soil exposed | 1 comorbid and more | no DM | HPT | no RF |
| 41-60 | Malay | Agricultural/Farming | Alive | Culture and Sensitivity | Soil exposed | 1 comorbid and more | DM | no HPT | no RF |
| 41-60 | BumiputeraOthers | Professionals | Death | Culture and Sensitivity | Soil exposed | No comorbid | no DM | no HPT | no RF |
| 41-60 | Malay | Unemployed | Death | Culture and Sensitivity | Non-soil exposed | No comorbid | no DM | no HPT | no RF |
| >60 | Malay | Unemployed | Alive | Culture and Sensitivity | Non-soil exposed | 1 comorbid and more | no DM | no HPT | no RF |
| 41-60 | Indian | Self-employed | Death | Culture and Sensitivity | Non-soil exposed | 1 comorbid and more | DM | HPT | no RF |
| 41-60 | Malay | Unemployed | Alive | Culture and Sensitivity | Soil exposed | No comorbid | no DM | no HPT | no RF |
| 41-60 | Malay | Unemployed | Alive | Culture and Sensitivity | Soil exposed | No comorbid | no DM | no HPT | no RF |
| 41-60 | Malay | Agricultural/Farming | Alive | Culture and Sensitivity | Soil exposed | No comorbid | no DM | no HPT | no RF |
| >60 | Malay | Agricultural/Farming | Alive | Culture and Sensitivity | Soil exposed | No comorbid | no DM | no HPT | no RF |
| 41-60 | Malay | Agricultural/Farming | Alive | Culture and Sensitivity | Soil exposed | 1 comorbid and more | DM | no HPT | no RF |
| 1-40 | Malay | Agricultural/Farming | Alive | Culture and Sensitivity | Soil exposed | 1 comorbid and more | DM | no HPT | no RF |
| 41-60 | Malay | Professionals | Alive | Culture and Sensitivity | Non-soil exposed | 1 comorbid and more | DM | no HPT | no RF |
| >60 | Malay | Agricultural/Farming | Alive | Culture and Sensitivity | Soil exposed | No comorbid | no DM | no HPT | no RF |
| >60 | Malay | Unemployed | Alive | Culture and Sensitivity | Soil exposed | No comorbid | no DM | no HPT | no RF |
| 41-60 | Malay | Self-employed | Alive | Culture and Sensitivity | Non-soil exposed | 1 comorbid and more | DM | no HPT | no RF |
| 41-60 | Malay | Unemployed | Alive | Culture and Sensitivity | Non-soil exposed | 1 comorbid and more | no DM | no HPT | no RF |
| >60 | Malay | Unemployed | Alive | Culture and Sensitivity | Non-soil exposed | 1 comorbid and more | no DM | no HPT | no RF |
| >60 | Malay | Unemployed | Alive | Culture and Sensitivity | Soil exposed | No comorbid | no DM | no HPT | no RF |
| 41-60 | Malay | Self-employed | Alive | Culture and Sensitivity | Soil exposed | 1 comorbid and more | no DM | no HPT | no RF |
| 1-40 | Malay | Self-employed | Alive | Culture and Sensitivity | Soil exposed | No comorbid | no DM | no HPT | no RF |
| 41-60 | Indian | Unemployed | Alive | Culture and Sensitivity | Non-soil exposed | 1 comorbid and more | no DM | no HPT | no RF |
| 41-60 | Indian | Factory/Industry | Alive | Culture and Sensitivity | Soil exposed | 1 comorbid and more | no DM | HPT | no RF |
| 41-60 | Malay | Factory/Industry | Alive | Culture and Sensitivity | Soil exposed | No comorbid | no DM | no HPT | no RF |
| >60 | Malay | Agricultural/Farming | Alive | Culture and Sensitivity | Soil exposed | 1 comorbid and more | DM | no HPT | no RF |
| 41-60 | Malay | Agricultural/Farming | Alive | Culture and Sensitivity | Soil exposed | No comorbid | no DM | no HPT | no RF |
| 41-60 | Malay | Agricultural/Farming | Alive | Culture and Sensitivity | Soil exposed | No comorbid | no DM | no HPT | no RF |
| >60 | Malay | Self-employed | Alive | Culture and Sensitivity | Soil exposed | No comorbid | no DM | no HPT | no RF |
| 41-60 | Malay | Unemployed | Death | Culture and Sensitivity | Soil exposed | 1 comorbid and more | DM | no HPT | no RF |
| 1-40 | Malay | Factory/Industry | Alive | Culture and Sensitivity | Soil exposed | No comorbid | no DM | no HPT | no RF |
| 41-60 | Malay | Unemployed | Alive | Culture and Sensitivity | Soil exposed | 1 comorbid and more | no DM | HPT | no RF |
| >60 | Malay | Professionals | Alive | Culture and Sensitivity | Non-soil exposed | 1 comorbid and more | DM | no HPT | no RF |
| 41-60 | Indian | Unemployed | Alive | Culture and Sensitivity | Non-soil exposed | 1 comorbid and more | no DM | no HPT | no RF |
| 41-60 | Malay | Self-employed | Alive | Culture and Sensitivity | Non-soil exposed | No comorbid | no DM | no HPT | no RF |
| 1-40 | Malay | Unemployed | Alive | Culture and Sensitivity | Non-soil exposed | 1 comorbid and more | DM | no HPT | no RF |
| 41-60 | Malay | Unemployed | Alive | Culture and Sensitivity | Soil exposed | No comorbid | no DM | no HPT | no RF |
| 41-60 | Malay | Agricultural/Farming | Alive | Culture and Sensitivity | Soil exposed | No comorbid | no DM | no HPT | no RF |
| 41-60 | Indian | Services | Alive | Culture and Sensitivity | Soil exposed | 1 comorbid and more | DM | no HPT | RF |
| >60 | Malay | Agricultural/Farming | Alive | Culture and Sensitivity | Soil exposed | No comorbid | no DM | no HPT | no RF |
| >60 | Malay | Agricultural/Farming | Alive | Culture and Sensitivity | Soil exposed | No comorbid | no DM | no HPT | no RF |
| 41-60 | Indian | Agricultural/Farming | Alive | Culture and Sensitivity | Soil exposed | No comorbid | no DM | no HPT | no RF |
| 41-60 | Malay | Agricultural/Farming | Alive | Culture and Sensitivity | Soil exposed | No comorbid | no DM | no HPT | no RF |
| 1-40 | Malay | Professionals | Alive | Culture and Sensitivity | Non-soil exposed | No comorbid | no DM | no HPT | no RF |
| >60 | Indian | Unemployed | Alive | Culture and Sensitivity | Soil exposed | No comorbid | no DM | no HPT | no RF |
| >60 | Malay | Professionals | Alive | Culture and Sensitivity | Soil exposed | No comorbid | no DM | no HPT | no RF |
| 1-40 | BumiputeraOthers | Factory/Industry | Death | Culture and Sensitivity | Soil exposed | No comorbid | no DM | no HPT | no RF |
| >60 | Malay | Agricultural/Farming | Death | Culture and Sensitivity | Soil exposed | 1 comorbid and more | DM | HPT | no RF |
| 41-60 | Malay | Agricultural/Farming | Death | Culture and Sensitivity | Soil exposed | 1 comorbid and more | DM | no HPT | no RF |
| >60 | Malay | Unemployed | Death | Culture and Sensitivity | Soil exposed | 1 comorbid and more | DM | HPT | no RF |
| 41-60 | Malay | Unemployed | Alive | Culture and Sensitivity | Non-soil exposed | No comorbid | no DM | no HPT | no RF |
| >60 | Malay | Unemployed | Alive | Culture and Sensitivity | Non-soil exposed | 1 comorbid and more | no DM | no HPT | no RF |
| 1-40 | Malay | Self-employed | Alive | Culture and Sensitivity | Non-soil exposed | No comorbid | no DM | no HPT | no RF |
| 1-40 | Malay | Self-employed | Death | Culture and Sensitivity | Soil exposed | No comorbid | no DM | no HPT | no RF |
| 1-40 | Malay | Unemployed | Alive | Culture and Sensitivity | Non-soil exposed | No comorbid | no DM | no HPT | no RF |
| 41-60 | Indian | Agricultural/Farming | Alive | Culture and Sensitivity | Soil exposed | 1 comorbid and more | DM | no HPT | no RF |
| 41-60 | Malay | Self-employed | Alive | Culture and Sensitivity | Soil exposed | 1 comorbid and more | no DM | no HPT | no RF |
| 41-60 | Malay | Unemployed | Death | Culture and Sensitivity | Soil exposed | 1 comorbid and more | DM | no HPT | no RF |
| >60 | Malay | Unemployed | Alive | Culture and Sensitivity | Non-soil exposed | 1 comorbid and more | DM | no HPT | no RF |
| 41-60 | Indian | Factory/Industry | Alive | Culture and Sensitivity | Non-soil exposed | 1 comorbid and more | no DM | no HPT | no RF |
| 41-60 | Malay | Self-employed | Death | Culture and Sensitivity | Soil exposed | 1 comorbid and more | DM | HPT | no RF |
| 41-60 | Indian | Agricultural/Farming | Alive | Culture and Sensitivity | Soil exposed | No comorbid | no DM | no HPT | no RF |
| 41-60 | Malay | Agricultural/Farming | Alive | Culture and Sensitivity | Soil exposed | No comorbid | no DM | no HPT | no RF |
| 41-60 | Malay | Unemployed | Alive | Culture and Sensitivity | Soil exposed | 1 comorbid and more | DM | no HPT | no RF |
| 1-40 | Malay | Unemployed | Alive | Culture and Sensitivity | Non-soil exposed | 1 comorbid and more | DM | HPT | no RF |
| 1-40 | Malay | Services | Alive | Culture and Sensitivity | Soil exposed | No comorbid | no DM | no HPT | no RF |
| 41-60 | Indian | Unemployed | Alive | Culture and Sensitivity | Non-soil exposed | 1 comorbid and more | DM | no HPT | no RF |
| >60 | Malay | Unemployed | Alive | Culture and Sensitivity | Soil exposed | No comorbid | no DM | no HPT | no RF |
| 41-60 | Malay | Unemployed | Alive | Culture and Sensitivity | Non-soil exposed | No comorbid | no DM | no HPT | no RF |
| 41-60 | Malay | Unemployed | Alive | Culture and Sensitivity | Soil exposed | No comorbid | no DM | no HPT | no RF |
| 41-60 | Malay | Agricultural/Farming | Alive | Culture and Sensitivity | Soil exposed | No comorbid | no DM | no HPT | no RF |
| 41-60 | Malay | Professionals | Alive | Culture and Sensitivity | Soil exposed | No comorbid | no DM | no HPT | no RF |
| 41-60 | Malay | Unemployed | Alive | Culture and Sensitivity | Soil exposed | No comorbid | no DM | no HPT | no RF |
| 41-60 | Chinese | Unemployed | Alive | Culture and Sensitivity | Soil exposed | No comorbid | no DM | no HPT | no RF |
| >60 | Indian | Unemployed | Alive | Culture and Sensitivity | Soil exposed | No comorbid | no DM | no HPT | no RF |
| 41-60 | Malay | Self-employed | Alive | Culture and Sensitivity | Soil exposed | No comorbid | no DM | no HPT | no RF |
| 41-60 | Malay | Unemployed | Alive | Serology | Soil exposed | No comorbid | no DM | no HPT | no RF |
| 41-60 | Malay | Unemployed | Alive | Culture and Sensitivity | Soil exposed | 1 comorbid and more | DM | no HPT | no RF |
| 41-60 | Chinese | Unemployed | Alive | Culture and Sensitivity | Soil exposed | 1 comorbid and more | no DM | HPT | no RF |
| 1-40 | Malay | Professionals | Alive | Culture and Sensitivity | Non-soil exposed | 1 comorbid and more | DM | no HPT | no RF |
| >60 | Malay | Unemployed | Alive | Culture and Sensitivity | Soil exposed | 1 comorbid and more | DM | no HPT | no RF |
| >60 | Chinese | Services | Alive | Culture and Sensitivity | Soil exposed | 1 comorbid and more | DM | HPT | no RF |
| 41-60 | BumiputeraOthers | Unemployed | Death | Culture and Sensitivity | Soil exposed | 1 comorbid and more | DM | HPT | no RF |
| >60 | Chinese | Unemployed | Alive | Culture and Sensitivity | Soil exposed | 1 comorbid and more | DM | HPT | no RF |
| >60 | Malay | Unemployed | Alive | Culture and Sensitivity | Soil exposed | No comorbid | no DM | no HPT | no RF |
| >60 | Malay | Unemployed | Alive | Culture and Sensitivity | Non-soil exposed | No comorbid | no DM | no HPT | no RF |
| 41-60 | Malay | Unemployed | Death | Culture and Sensitivity | Soil exposed | No comorbid | no DM | no HPT | no RF |
| 41-60 | Malay | Unemployed | Alive | Culture and Sensitivity | Soil exposed | 1 comorbid and more | DM | no HPT | no RF |
| 41-60 | Malay | Agricultural/Farming | Alive | Culture and Sensitivity | Soil exposed | No comorbid | no DM | no HPT | no RF |
| >60 | Malay | Unemployed | Alive | Culture and Sensitivity | Non-soil exposed | 1 comorbid and more | no DM | HPT | no RF |
| 1-40 | Indian | Agricultural/Farming | Alive | Culture and Sensitivity | Soil exposed | No comorbid | no DM | no HPT | no RF |
| 1-40 | Indian | Unemployed | Alive | Culture and Sensitivity | Soil exposed | 1 comorbid and more | DM | no HPT | no RF |
| >60 | Chinese | Professionals | Alive | Culture and Sensitivity | Soil exposed | 1 comorbid and more | no DM | no HPT | no RF |
| >60 | Malay | Unemployed | Alive | Culture and Sensitivity | Soil exposed | 1 comorbid and more | no DM | no HPT | no RF |
| 41-60 | Malay | Factory/Industry | Alive | Culture and Sensitivity | Soil exposed | 1 comorbid and more | DM | HPT | no RF |
| 41-60 | Malay | Factory/Industry | Alive | Culture and Sensitivity | Soil exposed | 1 comorbid and more | DM | no HPT | no RF |
| 1-40 | Malay | Factory/Industry | Alive | Culture and Sensitivity | Soil exposed | 1 comorbid and more | DM | no HPT | no RF |
| 41-60 | Malay | Agricultural/Farming | Alive | Culture and Sensitivity | Soil exposed | 1 comorbid and more | no DM | no HPT | no RF |
| >60 | Indian | Unemployed | Alive | Culture and Sensitivity | Soil exposed | 1 comorbid and more | DM | HPT | RF |
| 1-40 | BumiputeraOthers | Unemployed | Alive | Culture and Sensitivity | Soil exposed | No comorbid | no DM | no HPT | no RF |
| >60 | Malay | Unemployed | Alive | Culture and Sensitivity | Non-soil exposed | 1 comorbid and more | DM | HPT | RF |
| >60 | Malay | Self-employed | Alive | Culture and Sensitivity | Soil exposed | No comorbid | no DM | no HPT | no RF |
| 1-40 | Malay | Factory/Industry | Alive | Culture and Sensitivity | Soil exposed | 1 comorbid and more | DM | HPT | no RF |
| 1-40 | BumiputeraOthers | Professionals | Alive | Culture and Sensitivity | Soil exposed | No comorbid | no DM | no HPT | no RF |
| >60 | Malay | Unemployed | Alive | Culture and Sensitivity | Non-soil exposed | No comorbid | no DM | no HPT | no RF |
| 41-60 | Chinese | Unemployed | Alive | Culture and Sensitivity | Soil exposed | No comorbid | no DM | no HPT | no RF |
| 1-40 | Malay | Factory/Industry | Alive | Culture and Sensitivity | Soil exposed | No comorbid | no DM | no HPT | no RF |
| 1-40 | Indian | Unemployed | Alive | Culture and Sensitivity | Soil exposed | 1 comorbid and more | DM | HPT | no RF |
| 41-60 | BumiputeraOthers | Services | Death | Culture and Sensitivity | Soil exposed | 1 comorbid and more | DM | HPT | no RF |
| >60 | Indian | Unemployed | Death | Culture and Sensitivity | Non-soil exposed | 1 comorbid and more | DM | HPT | no RF |
| >60 | Malay | Unemployed | Alive | Culture and Sensitivity | Soil exposed | 1 comorbid and more | DM | HPT | no RF |
| 41-60 | Malay | Agricultural/Farming | Alive | Culture and Sensitivity | Soil exposed | 1 comorbid and more | DM | no HPT | no RF |
| 1-40 | Malay | Services | Alive | Culture and Sensitivity | Soil exposed | No comorbid | no DM | no HPT | no RF |
| 41-60 | Malay | Unemployed | Alive | Culture and Sensitivity | Soil exposed | No comorbid | no DM | no HPT | no RF |
| 1-40 | Malay | Professionals | Alive | Culture and Sensitivity | Soil exposed | No comorbid | no DM | no HPT | no RF |
| 41-60 | Malay | Unemployed | Alive | Culture and Sensitivity | Soil exposed | 1 comorbid and more | DM | no HPT | no RF |
| 1-40 | BumiputeraOthers | Professionals | Alive | Culture and Sensitivity | Soil exposed | 1 comorbid and more | DM | no HPT | no RF |
| 1-40 | Malay | Agricultural/Farming | Death | Culture and Sensitivity | Soil exposed | 1 comorbid and more | DM | no HPT | no RF |
| 41-60 | Malay | Agricultural/Farming | Death | Culture and Sensitivity | Soil exposed | 1 comorbid and more | DM | HPT | no RF |
| >60 | Malay | Unemployed | Alive | Culture and Sensitivity | Soil exposed | 1 comorbid and more | DM | no HPT | no RF |
| >60 | Chinese | Unemployed | Alive | Culture and Sensitivity | Soil exposed | 1 comorbid and more | no DM | no HPT | no RF |
| >60 | Malay | Unemployed | Alive | Culture and Sensitivity | Soil exposed | 1 comorbid and more | DM | HPT | no RF |
| >60 | Indian | Unemployed | Alive | Culture and Sensitivity | Soil exposed | No comorbid | no DM | no HPT | no RF |
| 41-60 | BumiputeraOthers | Professionals | Death | Culture and Sensitivity | Soil exposed | 1 comorbid and more | DM | HPT | no RF |
| 41-60 | Malay | Unemployed | Death | Culture and Sensitivity | Soil exposed | 1 comorbid and more | DM | no HPT | no RF |
| 41-60 | Malay | Unemployed | Alive | Culture and Sensitivity | Soil exposed | 1 comorbid and more | no DM | HPT | no RF |
| 41-60 | Malay | Agricultural/Farming | Alive | Culture and Sensitivity | Soil exposed | No comorbid | no DM | no HPT | no RF |
| 1-40 | Indian | Agricultural/Farming | Alive | Culture and Sensitivity | Soil exposed | No comorbid | no DM | no HPT | no RF |
| >60 | Malay | Unemployed | Alive | Culture and Sensitivity | Soil exposed | No comorbid | no DM | no HPT | no RF |
| 41-60 | Malay | Self-employed | Alive | Culture and Sensitivity | Non-soil exposed | No comorbid | no DM | no HPT | no RF |
| 41-60 | Malay | Unemployed | Alive | Culture and Sensitivity | Soil exposed | No comorbid | no DM | no HPT | no RF |
| 1-40 | Malay | Professionals | Alive | Culture and Sensitivity | Soil exposed | No comorbid | no DM | no HPT | no RF |
| 41-60 | Malay | Unemployed | Alive | Culture and Sensitivity | Soil exposed | 1 comorbid and more | DM | HPT | no RF |
| 41-60 | BumiputeraOthers | Unemployed | Alive | Culture and Sensitivity | Soil exposed | No comorbid | no DM | no HPT | no RF |
| >60 | Malay | Agricultural/Farming | Death | Culture and Sensitivity | Soil exposed | 1 comorbid and more | DM | HPT | no RF |
| 41-60 | Malay | Agricultural/Farming | Alive | Culture and Sensitivity | Soil exposed | 1 comorbid and more | DM | no HPT | no RF |
| 1-40 | Malay | Agricultural/Farming | Alive | Culture and Sensitivity | Soil exposed | No comorbid | DM | no HPT | no RF |
| >60 | Malay | Self-employed | Alive | Culture and Sensitivity | Soil exposed | 1 comorbid and more | DM | HPT | no RF |
| >60 | Malay | Unemployed | Alive | Culture and Sensitivity | Soil exposed | No comorbid | no DM | no HPT | no RF |
| 1-40 | Malay | Professionals | Death | Culture and Sensitivity | Non-soil exposed | No comorbid | no DM | no HPT | no RF |
| >60 | Malay | Self-employed | Alive | Culture and Sensitivity | Soil exposed | No comorbid | no DM | no HPT | no RF |
| >60 | Chinese | Agricultural/Farming | Alive | Culture and Sensitivity | Soil exposed | 1 comorbid and more | DM | no HPT | no RF |
| 1-40 | Malay | Factory/Industry | Alive | Culture and Sensitivity | Soil exposed | 1 comorbid and more | DM | no HPT | no RF |
| 41-60 | Indian | Services | Alive | Culture and Sensitivity | Non-soil exposed | No comorbid | no DM | no HPT | no RF |
| 41-60 | Indian | Agricultural/Farming | Death | Culture and Sensitivity | Soil exposed | No comorbid | no DM | no HPT | no RF |
| 41-60 | Malay | Agricultural/Farming | Alive | Culture and Sensitivity | Soil exposed | 1 comorbid and more | DM | no HPT | no RF |
| 41-60 | Malay | Agricultural/Farming | Alive | Culture and Sensitivity | Soil exposed | 1 comorbid and more | DM | no HPT | no RF |
